# Probing Light-Dependent Regulation of the Calvin Cycle Using a Multi-Omics Approach

**DOI:** 10.3389/fpls.2021.733122

**Published:** 2021-10-04

**Authors:** Nathaphon Yu King Hing, Uma K. Aryal, John A. Morgan

**Affiliations:** ^1^Davidson School of Chemical Engineering, Purdue University, West Lafayette, IN, United States; ^2^Purdue Proteomics Facility, Bindley Bioscience Center, Purdue University, West Lafayette, IN, United States; ^3^Department of Comparative Pathobiology, Purdue University College of Veterinary Medicine, West Lafayette, IN, United States; ^4^Department of Biochemistry, Purdue University, West Lafayette, IN, United States; ^5^Center for Plant Biology, Purdue University, West Lafayette, IN, United States

**Keywords:** isotopically non-stationary metabolic flux analysis, metabolite channeling, photoautotrophic metabolism, carbon fixation, cyanobacteria, proteomics, metabolic flux analysis (MFA)

## Abstract

Photoautotrophic microorganisms are increasingly explored for the conversion of atmospheric carbon dioxide into biomass and valuable products. The Calvin-Benson-Bassham (CBB) cycle is the primary metabolic pathway for net CO_2_ fixation within oxygenic photosynthetic organisms. The cyanobacteria, *Synechocystis* sp. PCC 6803, is a model organism for the study of photosynthesis and a platform for many metabolic engineering efforts. The CBB cycle is regulated by complex mechanisms including enzymatic abundance, intracellular metabolite concentrations, energetic cofactors and post-translational enzymatic modifications that depend on the external conditions such as the intensity and quality of light. However, the extent to which each of these mechanisms play a role under different light intensities remains unclear. In this work, we conducted non-targeted proteomics in tandem with isotopically non-stationary metabolic flux analysis (INST-MFA) at four different light intensities to determine the extent to which fluxes within the CBB cycle are controlled by enzymatic abundance. The correlation between specific enzyme abundances and their corresponding reaction fluxes is examined, revealing several enzymes with uncorrelated enzyme abundance and their corresponding flux, suggesting flux regulation by mechanisms other than enzyme abundance. Additionally, the kinetics of ^13^C labeling of CBB cycle intermediates and estimated inactive pool sizes varied significantly as a function of light intensity suggesting the presence of metabolite channeling, an additional method of flux regulation. These results highlight the importance of the diverse methods of regulation of CBB enzyme activity as a function of light intensity, and highlights the importance of considering these effects in future kinetic models.

## Introduction

The Calvin-Benson-Bassham (CBB) cycle is the primary metabolic pathway through which inorganic carbon is fixed by photosynthetic organisms (Bassham et al., 1954). However, the rate limiting steps and factors controlling the pathway are only partially captured by existing models (Arnold and Nikoloski, 2011; Jablonsky et al., 2011; Mills et al., 2020). Photosynthetic organisms are exposed to varying diurnal light conditions (Saha et al., 2016), as well as short term changes in light due to fluctuating weather patterns and shading (MacIntyre et al., 2000; Rascher and Nedbal, 2006), necessitating a complex regulatory structure that can deal with both short and long term changes in light availability. Varying enzymatic abundance is one way in which metabolic processes are regulated. Therefore, a thorough consideration of both the distribution of carbon fluxes and proteome-wide changes should result in a deeper understanding of photosynthetic behavior under different light intensities. Among photosynthetic organisms, cyanobacteria are interesting model organisms for their fast growth and their ability to be genetically transformed (Ducat et al., 2011). Cyanobacteria can serve as a way to transform atmospheric CO_2_ into useful end products (Carroll et al., 2018), such as astaxanthin (Diao et al., 2020), ethylene (Ungerer et al., 2012), ethanol (Dexter and Fu, 2009), or omega-3 fatty acids (Santos-Merino et al., 2018).

Flux of carbon through the CBB cycle is thought to be regulated at several levels: at the transcriptional level (Kusian and Bowien, 2006), at the substrate level, at the post-translational level by redox control (Michelet et al., 2013), or by carbonylation (e.g., RuBisCO activase). However, the regulation of the metabolic fluxes by light within the CBB cycle is still not completely understood. Some proteomic studies suggest enzymatic abundance of CBB cycle enzymes is a crucial metabolic lever (Battchikova et al., 2010; Jahn et al., 2018; Zavřel et al., 2019), while other studies indicate that the enzymes themselves undergo post-translational modifications that mediate enzymatic activity (Michelet et al., 2013; Tsukamoto et al., 2013; Slade et al., 2015; Tamoi and Shigeoka, 2015). Allosteric control of the CBB cycle (Zhu et al., 2013), CO_2_ limitations (Benschop et al., 2003; Tcherkez et al., 2008), and enzymatic localization (Agarwal et al., 2009; Long et al., 2018) have also been reported in cyanobacterial CBB cycle regulation. Differences in regulation are also present when comparing different photosynthetic organisms, such as those between plants, algae, and cyanobacteria (Kroth, 2015; Tamoi and Shigeoka, 2015). Evidently, CBB cycle activity is dependent on a complicated interplay of different methods of regulation. While some of these regulations are thought to play a role in metabolic adjustments to short term variations in light intensity, in this work we are interested in determining the relative abundance of Calvin cycle enzymes and the effects on CBB cycle fluxes when exposed to a constant light level. The use of proteomics and fluxomics together can help reveal the regulatory processes involved in the Calvin cycle. The use of untargeted proteomics allows us to examine dynamic changes in the entire proteome resulting from different light conditions. The use of fluxomics, specifically *via* the tracking of isotopic labeling in the form of isotopically non-stationary metabolic flux analysis (INST-MFA), allows for the characterization of *in vivo* fluxes of central metabolism in photosynthetic organisms (Young et al., 2011; Adebiyi et al., 2015). As has been shown in yeast and *E. coli*, combining these techniques allows for the inference of regulatory behavior that is not possible when relying on either technique alone (Hackett et al., 2016; Kochanowski et al., 2021).

## Results

### Carbon Fixation Rates and Calvin-Benson-Bassham Cycle Enzymatic Abundance in *Synechocystis* sp. PCC 6803 Are Light Dependent

**FIGURE 1:**
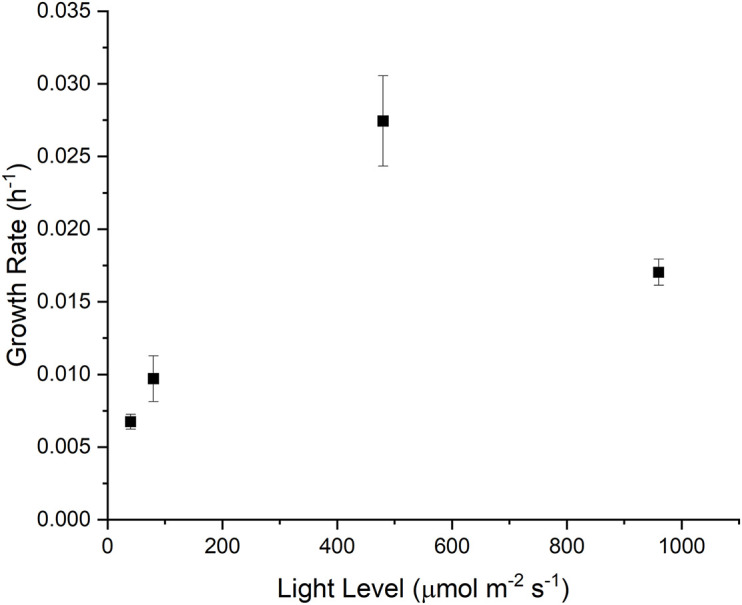
Measured growth rates (h^–1^) as a function of light level (μmol m^–2^ s^–1^).

To capture the phenotypical outcomes of *Synechocystis* sp. PCC 6803, we measured the cellular growth rate under four different light intensitites (40, 80, 480 and 960 μmol m^–2^ s^–1^), with the goal of capturing light-limited, light saturated and light-inhibited growth (Figure 1). Under light-limited conditions, growth of cyanobacteria is at least partially constrained by energy supply, while under light-inhibiting conditions, the production of radical oxygen species can cause photoinhibition or photobleaching in photoautotrophic organisms (Tripathy and Oelmüller, 2012; Hsieh et al., 2014).

Global non-targeted proteomics revealed distinct clustering (Supplementary Figure 1) under different light conditions, demonstrating significant global changes in the proteome. Three-dimensional principal component analysis was done on the proteomic data (Supplementary Figure 2), demonstrating distinct and reproducible clusters for each light condition.

**FIGURE 2:**
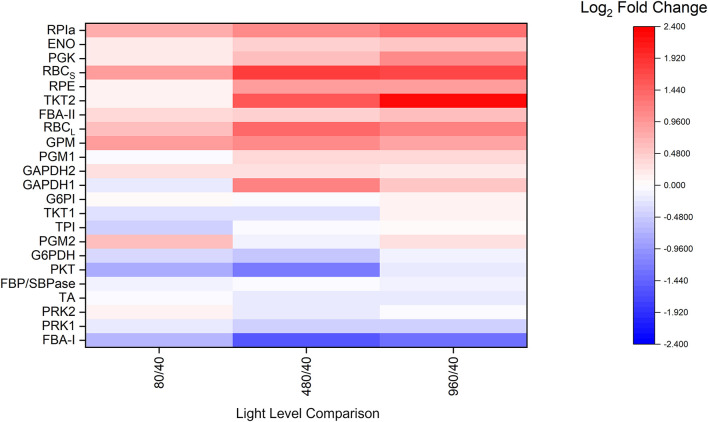
Average proteomic fold-change results relative to the 40 μmol m^–2^ s^–1^ condition for selected central carbon metabolism related enzymes, *n* = 3 for each light condition. ENO, enolase; FBA-I/II, fructose-bisphosphate aldolase class I/II; FBP/SBPase, D-fructose 1,6-bisphosphatase class 2/sedoheptulose 1,7-bisphosphatase; G6PDH, glucose-6-phosphate 1-dehydrogenase; G6PI, glucose-6-phosphate isomerase; GAPDH, glyceraldehyde 3-phosphate dehydrogenase; GPM, phosphoglucomutase; PGK, phosphoglycerate kinase; PGM, phosphoglycerate mutase; PKT, phosphoketolase; PRK, phosphoribulokinase; RBCl, ribulose bisphosphate carboxylase large chain; RBCs, ribulose bisphosphate carboxylase small subunit; RPE, ribulose-phosphate 3-epimerase; RPIa, Ribose-5-phosphate isomerase A; TA, transaldolase; TKT, transketolase; TPI, triose-phosphate isomerase. For isozymes, refer to NCBI accession numbers: GAPDH1 (CAA60134.1), GAPDH2 (CAA60135.1), PRK1 (P37101.1), PRK2 (WP_010873279.1), TKT1 (WP_010871940.1), TKT2 (WP_010874098.1), PGM1 (WP_010871279.1), and PGM2 (WP_010873603.1).

Several CBB cycle enzyme abundances are well correlated with light level (Figure 2). RPIa, ENO, PGK, RPE, TKT2, FBA-II, GPM, PGM, GAPDH1, and both subunits of RBC are largely positively associated with increased light levels and growth rate. In contrast, G6PDH, FBA-I, and PKT are negatively associated with light level and growth rate. Meanwhile, GAPDH2, G6PI, TKT, TPI, FBP/SBPase, TA, and both isoforms of phosphoribulokinase (PRK) do not change significantly over the studied light intensities. Additionally, the majority of detected abundances for photosystem subunits were negatively associated with light level, while TCA cycle enzyme abundances appeared to be largely unaffected (Supplementary Figure 3). These results demonstrate an individualized enzyme abundance response to changes in light level. Rather than simply increasing the overall enzyme abundance uniformly, this result is more consistent with a redistribution of enzyme abundances.

To further quantify the relationship between metabolic fluxes and individual enzyme abundances, the Pearson correlation coefficient and associated *p*-value was calculated for each enzyme and its corresponding reaction (Table 1). Based on these results, the enzymes ENO, GAPDH1, RBC_*L*_, PGM1, FBA-I, FBA-II, RPE, and RBC_*S*_ have the highest correlations with their corresponding flux. In cases where a relationship between enzyme abundance and corresponding flux could be observed (*p* < 0.1), a “pseudo-flux control coefficient” (PFCC), $P_{E_{i}}^{J}$, was also calculated.

**TABLE 1:** Calculated Pearson R^2^ and Pseudo-Flux Control Coefficients.

**Enzyme**	**Reaction**	**Pearson *R*^2a^**	***p* ^*b*^**	**$P_{E_{i}}^{J}$^*c*^**
ENO	2PGA → PEP	0.99	0.01	3.0
GAPDH1	3PGA ↔ GAP	0.98	0.02	1.6
RBC_*L*_	RUBP + CO2 → 3PGA + 3PGA	0.96	0.04	1.6
PGM1	3PGA ↔ 2PGA	0.95	0.05	3.3
FBA-I	DHAP + GAP ↔ FBP	0.94	0.06	–0.9
FBA-II	DHAP + GAP ↔ FBP	0.93	0.07	2.1
RPE	RU5P ↔ X5P	0.90	0.10	2.6
RBC_*S*_	RUBP + CO2 → 3PGA + 3PGA	0.90	0.10	1.1
TKT2	F6P ↔ E4P + EC2	0.85	0.15	
PRK1	RU5P → RUBP	0.85	0.15	
PRK2	RU5P → RUBP	0.83	0.17	
FBPase	FBP ↔ F6P	0.63	0.37	
RPIa	RU5P ↔ R5P	0.60	0.40	
PGK	3PGA ↔ GAP	0.51	0.49	
GAPDH2	3PGA ↔ GAP	0.50	0.50	
TKT2	S7P ↔ R5P + EC2	0.49	0.51	
G6PI	F6P ↔ G6P	0.47	0.53	
TPI	GAP ↔ DHAP	0.37	0.63	
TKT1	S7P ↔ R5P + EC2	0.36	0.64	
FBA-II	DHAP + E4P → SBP	0.24	0.76	
PGM2	S7P ↔ E4P + EC3	0.16	0.84	
SBPase	SBP → S7P	0.08	0.92	
TKT1	F6P ↔ E4P + EC2	0.04	0.96	

*^*a*^Pearson correlation coefficients between enzyme abundance and best-fit absolute value flux of the corresponding reaction.*

*^*b*^Associated *p*-value for the Pearson correlation coefficient test.*

*^*c*^Estimated pseudo-flux control coefficient (PFCC) for select enzymes.*


$$P_{E_{i}}^{J}=\frac{d\,J}{d\,E_{i}}\,\frac{E_{i}}{J}$$

In metabolic control analysis, a flux control coefficient, $C_{E_{i}}^{J}$, is the scaled infinitesimal change of flux, J, in response to some infinitesimal change in enzyme level, E_*i*_, around a reference state (Ap Rees and Hill, 1994). Typically, a flux control coefficient is most accurate when a minimal number of enzymes are perturbed and other enzyme abundances are held constant to minimize deviations from linearity, a quality that is not met in these experimental conditions. Furthermore, due to a scarcity of data points, and because these experimental perturbations are not infinitesimal, these coefficient estimates are likely to be inaccurate (Pettersson, 1996; Wu et al., 2004; Link and Weuster-Botz, 2007), and do not abide by the summation or connectivity theorems typically applied in metabolic control analysis. As a result, we relied on an inexact approximation for the FCC, the PFCC, where these requirements are relaxed. PFCCs can be a useful way to determine if there is a strong relationship between flux and enzyme abundance around a reference state. In this case, the reference state was chosen to be the 80 μmol m^–2^ s^–1^ steady state. The non-linear fitting method (Small, 1993) was used to estimate the coefficients for each enzyme. In prior literature, enzymes with high flux control coefficients have been suggested as potential targets for overexpression (Fell, 1998), as they potentially offer high positive control over a pathway. Of the selected enzymes, PGM1, ENO, RPE, and FBA-II enzymes have the highest PFCCs, with FBA-II and both RBC subunits in particular corroborating experimental results which demonstrate enhanced growth rates in *Synechocystis* (Liang and Lindblad, 2016). Interestingly, these PFCCs were calculated to be above unity. In such cases, the flux increases proportionally more than the enzymatic abundance around the reference state, indicating the presence of other confounding variables that positively affect flux. For instance, substrate concentrations may be altered after perturbation of light level, altering flux. Additionally, CBB cycle enzymes are known to adopt more active conformations as a result of redox-sensitive post-translational modifications (Michelet et al., 2013), meaning that the same amount of enzyme concentration can support a larger flux under favorable reducing conditions. The flux control coefficients were not calculated for enzymes where the correlation between enzyme abundance and flux were ambiguous or negligible (Table 1, *p* > 0.1), as it was not possible to perform any accurate non-linear fitting between *J* and *E*.

### Variation in Steady State ^13^C Enrichment of Calvin-Benson-Bassham Cycle Metabolites Under Different Light Conditions Implies a Light-Dependent Cytosolic Pool Fraction

Previous isotopic labeling studies (Huege et al., 2011; Young et al., 2011) found notable ^13^C labeling kinetics wherein downstream metabolites had higher ^13^C labeling than their precursors at the steady state condition. A proposed explanation for this phenomenon is that CBB cycle metabolites may be subjected to metabolite channeling, a phenomenon where enzymes are organized into complexes that pass metabolites to each other, thereby potentially increasing the local concentration resulting in higher enzymatic reaction rates (Süss et al., 1993; Anderson et al., 2005; Abernathy et al., 2017a). The most obvious example of metabolite channeling in cyanobacteria occurs in the carboxysome, where RuBisCO and carbonic anhydrase are localized (Yu et al., 1994; Rae et al., 2013; Long et al., 2018; Abernathy et al., 2019) to facilitate carbon fixation by raising the local concentration of CO_2_ around RuBisCO.

**FIGURE 3:**
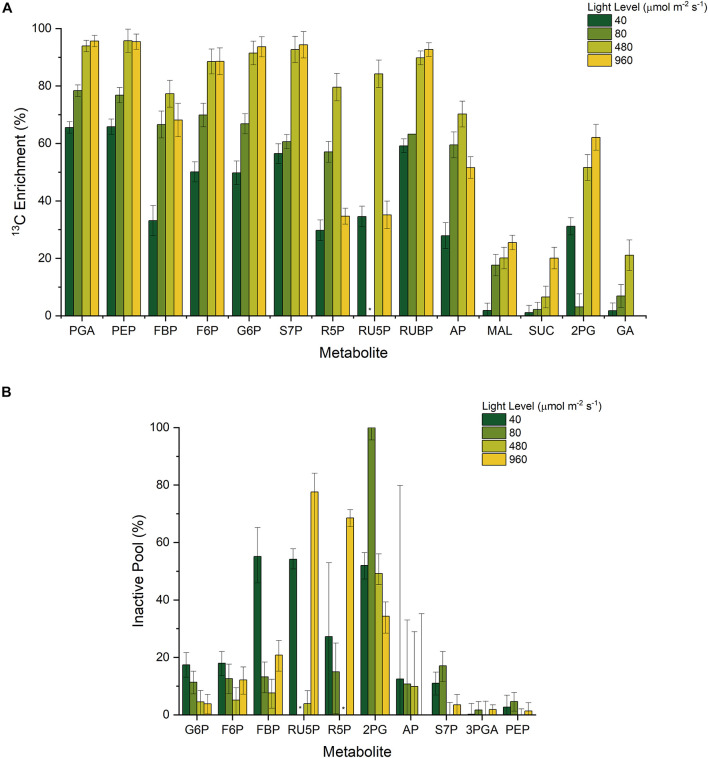
**(A)** Measured ^13^C enrichment 20 min after initial NaH^13^CO_3_ pulse and **(B)** Estimated inactive pool sizes from INST-MFA; * n.d.

Our results reinforce these observations, as we consistently found lower ^13^C labeling enrichments in the first of sequential pairs of metabolites such as FBP and F6P, as well as RU5P and RUBP (Figure 3A). The presence of metabolite channeling would result in a more quickly labeled ‘active’ pool as well as a slowly labeled ‘inactive’ pool that dilutes the overall labeling enrichment measurement. With the incorporation of dilution parameters, it is also possible to estimate the fraction of active and inactive intermediates using INST-MFA (Figure 3B) (Ma et al., 2014). In general, the inactive pool fractions were minimal during the highest growth condition (480 μmol m^–2^ s^–1^), and significantly higher when light was either limited or inhibiting growth.

### Carbon Flux Distribution Across Light Regimes

For the INST-MFA model, we designated the metabolic network to encompass reactions in the CBB cycle, TCA cycle, photorespiration, and the phosphoketolase shunt (Supplementary File 2). Although biomass composition may be altered slightly across light levels (Zavřel et al., 2019), these differences are not statistically significant at the measured growth rates, and we failed to identify statistically significant differences in our own carbohydrate and protein assays. Therefore, an additional simplifying assumption was made that biomass composition is not significantly altered for WT cells grown under varying light levels. The net carbon fixation was computed from the overall growth rate, allowing for the individual fluxes to be represented on an absolute basis in units of μmol C gDW^–1^ h^–1^ (assuming 0.0412 mol C gDW^–1^ based on the WT biomass equation). In all tested conditions, as expected, the majority of carbon (∼95%) was fixed from RuBisCO activity as opposed to other CO_2_ incorporating reactions such as PEP carboxylase. In general, it is difficult to statistically differentiate between small CBB cycle fluxes due to relatively large error bounds (Supplementary File 2). This indicates that relative carbon flux can be constantly regulated to partition itself into different biomass components regardless of the light level.

A relatively small amount of oxidative pentose phosphate pathway flux is observed under light-limited conditions, which results in differences in isotopic labeling of R5P and RU5P. However, this activity is nearly zero at light saturating or light inhibiting conditions, corroborating prior observations of efficient photoautotrophic metabolism (Ueda et al., 2018).

**FIGURE 4:**
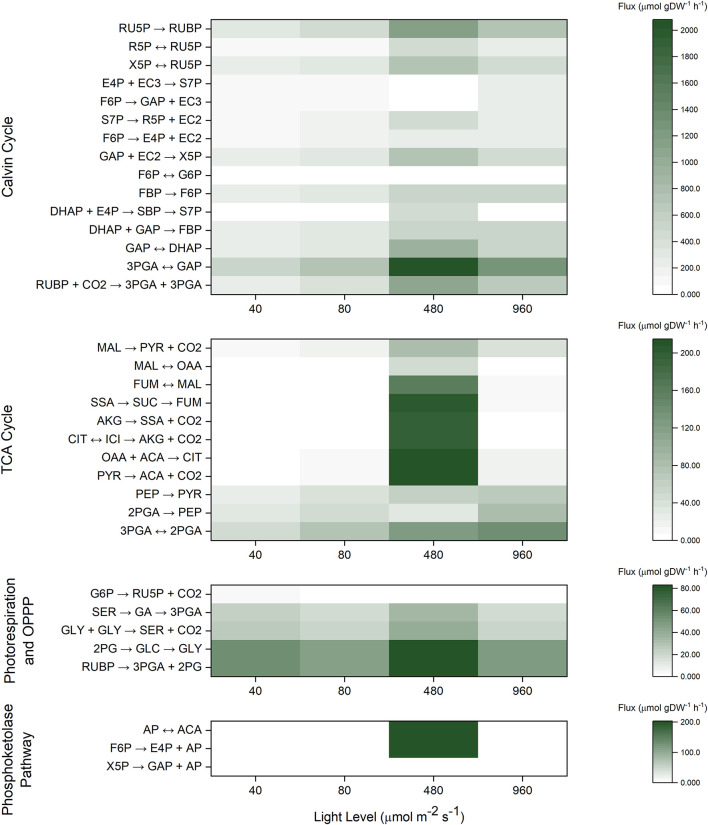
Heat maps for INST-MFA predicted best-fit absolute flux values (μmol gDW^–1^ h^–1^) across all four light levels.

Calculating fluxes for the TCA cycle precisely is difficult due to its comparatively low values under photoautotrophic conditions (Ma et al., 2014), resulting in no statistically significant differences in TCA cycle flux between all light intensities (Supplementary File 2). On the other hand, there is a generally slight increase in the ^13^C labeling of TCA cycle intermediates malate and succinate as the light level increases, which is consistent with an increase in TCA cycle flux. Similarly, a slight increase in labeling of the photorespiration intermediates 2PG and GA is observed at saturating and light inhibiting conditions, but photorespiration fluxes are again poorly resolved due to its relatively low values as compared to the CBB cycle. Such a result indirectly supports the notion that photorespiration may serve as a way to protect against oxidative stress (Sunil et al., 2019). We note that predicted photorespiration activity has non-zero lower bounds even under low light conditions (Figure 4), corroborating prior literature (Burnap et al., 2015; Klemke et al., 2015). Furthermore, as shown in flux maps (Abernathy et al., 2017b) and in previous genome scale modeling (Nogales et al., 2012), photorespiration can be beneficial for high light photosynthesis, and was also found to play an important role during maximal *Synechococcus elongatus* UTEX 2973 growth (Abernathy et al., 2017b), despite carbon loss.

## Discussion

### The Abundance of Calvin-Benson-Bassham Cycle Enzymes in *Synechocystis* sp. PCC 6803 Are Variably Dependent on Light Level

Although it has been shown that the total protein concentration of cyanobacteria is relatively invariant (Zheng and O’Shea, 2017), individual abundances of light harvesting complexes (LHC) and metabolic enzymes as revealed by proteomic studies are at least partially dependent on light level. Specifically, the relative abundances of LHC proteins detected in this study generally decreased with increasing light level, in agreement with previous proteomic studies (Jahn et al., 2018; Zavřel et al., 2019). In contrast, many Calvin cycle enzymes generally increased with increasing light level. For example, the amount of RPIa, ENO, PGK, both subunits of RBC, RPE, TKT2, FBA-II, GPM, PGM, and GAPDH1 are largely positively associated with increased light levels and growth rate, which is the expected behavior in a light dependent carbon fixation pathway like the Calvin cycle (Figure 2). On the other hand, enzymes such as G6PDH, FBA-I, and PKT are also notable for being negatively associated with light level and growth rate, but these results are not surprising. G6PDH, as the first step of the oxidative pentose phosphate pathway (OPPP), is known to be more active in the dark (Ueda et al., 2018). *Synechocystis* contains two FBA isozymes (Class I and Class II) (Nakahara, 2003), of which FBA-II is thought to be the more active and abundant enzyme. Additionally, phosphoketolase (PKT) showed an inversely correlated expression profile compared to the majority of CBB cycle enzymes. Previously, PKT was found to be non-essential, but slightly beneficial to growth rate during photoautotrophic conditions (Xiong et al., 2015). PKT catalyzes an alternate entry point into the tricarboxylic acid (TCA) cycle. Interestingly, phosphoglycerate mutase (PGM) and enolase (ENO) do not share this behavior, despite also catalyzing an entry point into the TCA cycle. It was earlier speculated that PKT may be able to improve the carbon efficiency since the phosphoketolase pathway does not lose carbon but utilizes different amounts of cofactors (Xiong et al., 2015). The balance between the phosphoketolase-dependent pathway and the phosphoketolase-independent pathway may perhaps reflect a different balance of energetic cofactors under different light levels. These results also indicate that some steady-state enzyme abundances are more light-dependent than others despite being part of the same pathway, suggesting possible differences in regulatory behavior. These differences in sensitivity between individual CBB cycle enzymes and light level were not fully apparent when grouping enzymes, as was done in previous proteomic studies (Jahn et al., 2018; Zavřel et al., 2019).

Although integrating proteomic and fluxomic datasets has been previously discussed in the literature (Zhang et al., 2010; Winter and Krömer, 2013), actual studies incorporating these techniques together remain uncommon. Prior studies (Saha et al., 2016; Nakajima et al., 2017) examined transcriptomic results together with fluxes to describe regulation in *Synechocystis* 6803, but the use of proteomics over transcriptomics represents an iterative improvement. The use of proteomics and fluxomics together presents an opportunity to examine correlations between fluxes and enzyme abundances. Based on statistical correlation analysis (Table 1), many enzyme abundances appear to be uncorrelated with their actual corresponding flux. There may be several reasons for the apparent insensitivity of the abundance of enzymes like GAPDH2, G6PI, TKT, TPI, FBP/SBPase, TA, and both isoforms of PRK to light intensity. First, the isomerases (G6PI and TPI) have generally been considered poor candidates of exerting control over the pathway based on kinetic parameters (Zhu et al., 2007), having relatively rapid kinetics. The relatively insensitive behavior of GAPDH2 is also not unexpected when considering that the more photosynthetically active GAPDH1 isoform is positively associated with growth. Another possible contributing reason may be due to a slow protein turnover rate, which implicates post-translational regulation as a way to control flux (Karlsen et al., 2021).

As $\frac{d\,J}{d\,E_{i}}$ can vary widely around different reference states, the analysis of the relationship between enzyme abundance and flux can be complicated. One interpretation of uncorrelated proteomic and fluxomic data is that these reactions are regulated at a post-translational level (Bro et al., 2003). These results appear to contradict previous estimates of CBB cycle enzymes with higher FCCs as derived from kinetic models (Janasch et al., 2019; Wu et al., 2020) especially for PRK and FBP/SBPase, strongly implying that the control of these fluxes, in wild-type, are not based on enzyme abundances. One explanation may be that the overexpression of these enzymes is conditionally beneficial to growth depending on the light condition due to different FCCs at different reference states. Indeed, our prior study indicated significant growth advantages when FBP/SBPase was overexpressed in *Synechocystis* 6803 at low light conditions, but a much smaller advantage when the light level was increased (Hing et al., 2019). Some alternative forms of regulation that could explain the changes in fluxes are discussed in the section below.

### Thylakoid Membrane Localization of Calvin-Benson-Bassham Cycle Enzymes Can Provide a Basis for Metabolite Channeling

There is compelling evidence of localization of CBB cycle enzymes near the thylakoid membrane (Dani and Sainis, 2005; Agarwal et al., 2009). Specifically, RPI, PRK, RuBisCO, PGK, and GAPDH, which comprise five sequential enzymes of the CBB cycle were found to be preferentially, but not completely, localized near the thylakoid membranes in *Synechocystis* sp. PCC 6803 (Süss et al., 1993; Dani and Sainis, 2005; Agarwal et al., 2009). Similarly, aldolase, SBPase, and FBPase have been noted to be preferentially localized around the thylakoid membrane in pea leaves (Alscher-Herman, 1982; Hermoso et al., 1989; Andres et al., 1990; Anderson et al., 2005) even though these enzymes have functional activity in soluble *in vitro* assays devoid of any membranes. These enzymes can benefit from the production of energetic cofactors from the light reactions of photosynthesis occurring around the thylakoid membrane, and are able to quickly interact with the thioredoxin-ferredoxin system to transition between their redox regulated post-translational states (Michelet et al., 2013). In the aforementioned immunolabeling studies, it was demonstrated that at least a fraction of the detected CBB enzymes were cytosol localized, although an explanation for the partial split between cytosol and thylakoid membrane localized fractions was not explicitly presented therein. Yet, as shown previously in chloroplasts, exposure to sufficient quantities of light and/or reducing factors can induce a release of enzymes from the thylakoid membranes (Ben-Bassat and Anderson, 1981; Tanaka et al., 1990), suggesting a strong link between redox environment and enzyme localization.

Consequently, it is reasonable to conclude that the co-localization of CBB cycle enzymes onto the periphery of the thylakoid membranes can result in the observed ^13^C enrichment pattern, by forming a metabolite channel. The formation of a metabolite channel can regulate flux for several reasons. First, the local concentration of metabolites can be higher than the overall measured intracellular concentration. Second, the kinetic parameters of thylakoid-bound enzymes can be significantly different from their soluble isoforms. Our data indicates that while metabolite channeling is generally occurring under photoautotrophic conditions, the fractional size of the inactive pools is not constant across all conditions. Most notably, the predicted fractional inactive pool size of RU5P is significantly higher at the light-inhibiting condition and to a lesser extent under the low light conditions as opposed to the optimal light condition because of a stark difference in RU5P enrichment (0.84 and 0.35 at 480 and 960 μmol m^–2^ s^–1^, respectively) between those conditions despite a similarly labeled RUBP (0.90 and 0.93 at 480 and 960 μmol m^–2^ s^–1^, respectively) (Figure 3). To a lesser extent, this trend is also present for FBP and F6P enrichment. This result implies a movement of PRK and FBP/SBPase to the cytosol to turn over the inactive cytosolic pool at highly reducing conditions. Thus, we infer from this data that the inactive pool sizes are related to the redox environment of the cell. During light inhibiting conditions, the production of radical oxygen species can alter redox balance to favor oxidation (Hsieh et al., 2014). Similarly, the lower amount of reducing cofactors produced during low light conditions also results in a non-optimal redox balance. It is only around the optimal light condition where redox conditions are ideal that PRK and FBP/SBPase most effectively turns over the pool of their respective substrates.

Recently, a synthetic metabolite channel was engineered in *Bacillus subtilus* to enhance N-acetylglucosamine synthesis (Lv et al., 2020), which provides concrete empirical evidence that metabolite channeling can significantly alter reaction efficiency. The creation of metabolite channels is a promising metabolic engineering strategy because it is orthogonal to many common metabolic engineering strategies such as enzyme overexpressions or knockdowns, allowing for a more complex or fine-tuned approach. While the exact mechanism remains unclear, it is apparent that the fraction of the inactive pool was minimized under high growth rates, and therefore may provide a future target for metabolic engineering. For example, addition of a thylakoid-binding domain to other enzymes outside the CBB cycle to make use of channeled metabolites can potentially increase the rate of specific reactions outside of the high growth rate condition. Both the proteomic and labeling data in combination with prior literature suggest a complex regulatory system where light availability simultaneously influences enzyme activity and enzyme localization, as has already been known to occur with carboxysomes (Sun et al., 2016), resulting in control of CBB fluxes. Efforts to capture these important regulatory mechanisms with mechanistic kinetic models should begin to consider these previously neglected elements, as thylakoid-bound enzymes may have different kinetic parameters than their unbound forms.

### Formation of the Phosphoribulokinase/CP12/Glyceraldehyde 3-Phosphate Dehydrogenase Complex as an Additional Method of Calvin-Benson-Bassham Cycle Regulation

Phosphoribulokinase is known to form a complex together with glyceraldehyde-3-phosphate dehydrogenase (GAPDH) and the small protein CP12, forming a multi-enzyme complex that appears to be conserved in both cyanobacteria and plants (Trost et al., 2006; Marri et al., 2010; López-Calcagno et al., 2014). This complex is also known to form under an oxidative redox environment (Trost et al., 2006), which inhibits both PRK and GAPDH activity, and dissociates under highly reducing conditions. In *Synechocystis*, two GAPDH genes exist, *gap1* and *gap2*, of which *gap2* appears to be responsible for anabolic CBB cycle activity, and *gap1* for catabolic glycolytic activity (Koksharova et al., 1998). Of these genes, *gap2* appears to share sequence similarity with chloroplastic *gapAB* genes which constitutes the PRK/CP12/GAPDH complex in higher plants (Koksharova et al., 1998). Curiously, our proteomic study suggested that both PRK and GAPDH2 (*gap2*) expression was relatively insensitive to light level, a result corroborated in a recent proteomic study (Zavřel et al., 2019). This contrasts with many other CBB cycle enzymes, which are positively correlated with light-intensity. Therefore, these results suggest that the formation of the PRK/CP12/GAPDH is a significant way that cells regulate PRK and GAPDH activity as opposed to simply varying enzymatic abundance. One advantage of forming such a complex is that the formation or dissociation of this complex is much more rapid than *de novo* synthesis or degradation of proteins (Howard et al., 2008).

**FIGURE 5:**
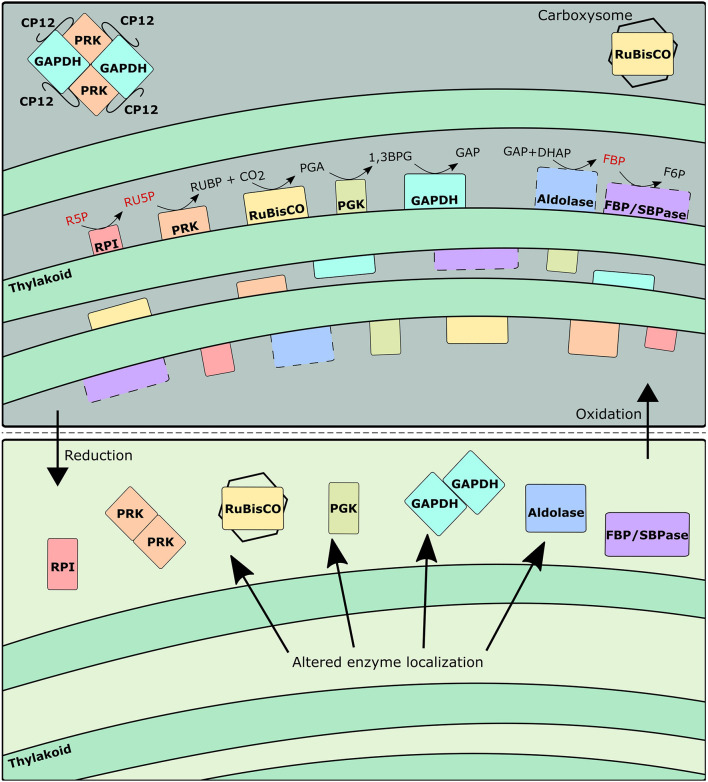
Schematic representing changes to enzyme localization and grouping as a function of redox environment. Dashed lines indicate enzymes not yet confirmed to be thylakoid-bound in *Synechocystis*. Metabolites in red indicate particularly large inactive pool fractions during oxidative conditions.

During light-inhibiting conditions, the production of radical oxygen species becomes significant and may promote PRK/CP12/GAPDH formation similarly to a low light or dark condition as reflected by the lower growth rate. Therefore, under light-inhibiting conditions where oxidative stress could be high, the formation of PRK/CP12/GAPDH complex can be an additional way to explain the reduced flux through PRK and GAPDH. The PRK/CP12/GAPDH complex has much lower activity as compared to the dimeric PRK or GAPDH alone (Howard et al., 2008). Based on these findings, both the formation of metabolic channels and the formation of the PRK/CP12/GAPDH complex are coordinated by the redox environment (Figure 5).

### Inactive Pool Sizes Are a Metabolic Lever for Controlling Flux Throughout the Calvin-Benson-Bassham Cycle

It was not possible to resolve any statistically significant differences in the relative carbon flux distribution throughout the CBB cycle under different light conditions because of the large upper and lower bounds, implying a strict level of regulation inherent in *Synechocystis* central metabolism. In comparison with earlier INST-MFA flux maps performed under several different light conditions (Young et al., 2011; Jazmin et al., 2017; Hing et al., 2019), these error bounds were much larger, likely due to the addition of more inactive pools in the stoichiometric model. This contrasts with the estimated active and inactive fractional pool sizes of the metabolites themselves, which are frequently significantly different (Figure 3). In other words, the CBB cycle can regulate carbon flow to maintain biomass growth, under photoautotrophic conditions, at least in part *via* modulating the fractional size of the active intermediate metabolite concentrations. Small changes in inactive pool sizes can result in large differences in absolute flux. Recent metabolomic studies confirm significant changes in intermediates across the diurnal cycle to maximize growth during the day (Werner et al., 2019; Jaiswal and Wangikar, 2020). The ability to modulate the fractional size of active intermediates during different light conditions provide an additional means to control the rate of CBB cycle reactions.

## Conclusion

The CBB cycle is a complex network of reactions whose regulation is partially dependent on many contributing factors. While the abundances of many CBB cycle enzymes are well correlated with light levels, other enzymes such as both PRK isozymes, FBP/SBPase, and GAPDH2 are not, suggesting the importance of additional forms of regulation. Our ^13^C labeling data taken across multiple light levels suggest that metabolite channeling is not only present but plays an important role in modulating specific reaction fluxes, as revealed by the presence of inactive metabolite pools. Cyanobacteria possess the ability to localize CBB cycle enzymes around the thylakoid membrane, potentially resulting in altered reaction kinetics and changes to the active pool sizes of CBB cycle intermediates. Slight changes in the estimated active pool sizes can result in large changes in predicted reaction fluxes in INST-MFA. The localization of enzymes therefore represents an additional lever that could be used to optimize reaction rates within or around the CBB cycle. This research provides an example of a combined multi-omic approach to characterize metabolic changes in response to altered environmental conditions. In particular, the use of fluxomics and proteomics can reveal coordinated *in vivo* regulatory mechanisms like metabolite channeling that may not be observable when isolated enzymes are characterized *in vitro*. On the other hand, metabolite channeling is only one of several methods of flux regulation available to cyanobacteria, and the rate at which enzymes can transition between different subcellular locations is unclear. Additional research regarding metabolite channeling as it occurs in cyanobacteria, especially during diurnal conditions, may reveal additional insight about the extent to which it plays a role in regulating fluxes.

## Materials and Methods

### Cultivation Conditions

*Synechocystis* sp. PCC 6803 cells were cultured in batch in BG-11 PC media (van Alphen et al., 2018) with no organic carbon source in a photobioreactor (PBR101, Phenometrics, MI, United States) at 30°C, with a magnetic stir bar (300 rpm) and 600 mL total volume with constant sparging of air (1 L/min). Continuous light at four different intensities (40, 80, 480 and 960 μmol m^–2^ s^–1^) was provided by a cold LED light (PBR101, Phenometrics, MI). The light spectra is provided as a Supplementary Figure 4. Optical density at 730 nm was measured to provide cell density estimates at each condition, and the growth rate was estimated using the Levenberg Marquardt algorithm during early exponential growth phase, ranging from OD_730_ 0.1 to 0.7. ^13^C was introduced to the bioreactor after OD_730_ reached at least 0.6.

### Isotopically Non-stationary Metabolic Flux Analysis

Isotopically non-stationary metabolic flux analysis (INST-MFA) allows for estimation of flux distribution throughout photoautotrophic central carbon metabolism by tracking the transient incorporation of ^13^C into cellular metabolism (Ma et al., 2014, 2017; Abernathy et al., 2017b; Hendry et al., 2017; Gopalakrishnan et al., 2018). NaH^13^CO_3_ in water was injected into the bioreactor to a total concentration of 1 g NaH^13^CO_3_/L to quickly introduce ^13^C into cellular metabolism during exponential phase. Culture samples (10 mL) were removed and quenched at separate time intervals (0, 0.5, 1, 1.5, 2, 3, 5, 10, and 20 min) into 40 mL pre-chilled 60% methanol in a dry ice-ethanol bath and rapidly pelleted at 8000 g, −20°C and then extracted. The MATLAB-based software INCA v1.8 (Young, 2014) was used to estimate pathway fluxes at each light condition (Supplementary File 2). Flux estimates were only accepted if the calculated χ^2^ goodness of fit is within the expected 95% confidence interval. Upper and lower bounds on fluxes were estimated using the parameter continuation method.

### Extraction of Metabolites and LC-MS/MS

Pellets were extracted using 500 μL chloroform/methanol (3:7 v/v) under −20 °C for 2 h followed by two rounds of 500 μL 0.2 M NH_4_OH (Prasannan et al., 2018). The upper methanol-water layer was collected. Samples were dried under N_2_ and then reconstituted in 60 μL ultrapure water. An QTRAP 5500 (AB Sciex) linked to HPLC (Shimadzu) was used to identify central carbon metabolites using an established ion-pairing method (Shastri and Morgan, 2007; Ma et al., 2017) using a Polaris C18 column (150 × 2 mm) (Agilent, CA, United States).

### Sample Preparation for Proteomic Analysis

Immediately prior to labeling experiments, a 20 mL aliquot of culture was withdrawn and pelleted for use in non-targeted proteomics. Cell pellets were washed twice in 20 mM PBS and then stored at −80°C until sample preparation for proteomic analysis. Cell pellets were re-suspended in 400 μL of 100 mM ammonium bicarbonate containing 1 mM phenylmethylsulfonyl fluoride and homogenized at high pressure (20,000 psi) at 6500 rpm for 90 s (Bertin Technologies SAS). Homogenate was transferred to new tubes and centrifuged at 13,500 rpm for 15 min at 4°C. The supernatant was transferred to a new tube and treated as a soluble fraction, and the pellets were treated as insoluble fraction. Proteins in the soluble fractions were precipitated overnight at −20°C using 4 volume of cold (−20°C) acetone and precipitated proteins were pelleted by centrifugation at 13,500 rpm for 15 min at 4°C. The soluble and insoluble fractions were dissolved in 8 M urea at room temperature for 1 h with continuous vortexing. The protein concentration was determined by Bicinchoninic Acid BCA assay and 50 μg of the protein (equivalent volume) was used for proteomics sample preparation. Samples were incubated in 10 mM dithiothreitol at 37°C for an hour for reduction followed by incubation in 20 mM iodoacetamide for an hour at room temperature in the dark for alkylation. Samples were digested using Trypsin/LysC protease mix at 1:25 (enzyme to substrate) ratio at 37°C. After 3 h of digestion, additional Trypsin/LysC protease mix was added at 1:50 (enzyme to substrate) ratio and digestion was allowed to proceed overnight at 37°C. Peptides were desalted using C18 micro spin desalting columns (The Nest Group, Inc.). Eluted peptides were dried and re-suspended in 3% acetonitrile/0.1% formic acid to a final concentration of 1 μg/μL and 1 μL (1 μg) was loaded used for LC-MS/MS analysis.

### LC-MS/MS Analysis for Peptide Sequencing

Peptides were analyzed in an Dionex UltiMate 3000 RSLC nano System coupled on-line to Q Exactive Orbitrap High Field Hybrid Quadrupole Mass Spectrometer (Thermo Fisher Scientific, Waltham, MA, United States) as described previously (Aryal et al., 2018; Mohallem and Aryal, 2020). Briefly, reverse phase peptide separation was accomplished using a trap column (300 μm ID × 5 mm) packed with 5 mm 100 Å PepMap C18 medium coupled to a 50-cm long × 75 μm inner diameter analytical column packed with 2 μm 100 Å PepMap C18 silica (Thermo Fisher Scientific). The column temperature was maintained at 50°C. Sample was loaded to the trap column at a flow rate of 5 μL/min and eluted from the analytical column at a flow rate of 300 nL/min using a 120-min LC gradient. The column was washed and equilibrated by using three 30 min LC gradient before injecting next sample. Precursor ion (MS1) scans were collected at a resolution of 120,000 and MS/MS scans at a resolution of 15,000 at 200 m/z in data dependent acquisition mode.

### LC-MS Data Analysis

LC-MS/MS data were analyzed using MaxQuant (version 1.6.3.4) against the *Synechocystis* sp. PCC 6803 database downloaded from the NCBI^1^. We edited the following parameters for our search: precursor mass tolerance of 10 ppm; enzyme specificity of trypsin/Lys-C enzyme allowing up to 2 missed cleavages; oxidation of methionine (M) as a variable modification and carbamidomethylation (C) as a fixed modification. False discovery rate (FDR) of peptide spectral match (PSM) and protein identification was set to 0.01. Proteins with LFQ >0 and MS/MS (spectral counts) ≥2 were considered as true identification and used for further downstream analysis. In total, 1,202 proteins were detected in at least one of the four tested conditions.

To compare data between different light conditions, the label free quantification (LFQ) intensities were used. The intensities of the soluble and insoluble fractions were combined for each replicate. InfernoRDN^2^ (Polpitiya et al., 2008) was used to perform a Kruskal-Walis test to determine *p*-values and associated *q*-values to correct for background false discovery rate for the 294 detected proteins that were detected at least twice per triplicate at all conditions (Supplementary File 8).

## Data Availability Statement

The original contributions presented in the study are publicly available. This data can be found here: the MassIVE repository (https://massive.ucsd.edu/ProteoSAFe/static/massive.jsp), under accession number MSV000087721.

## Author Contributions

NY designed and conducted the isotopic labeling experiments, analyzed all the data, and wrote the manuscript. UA conducted the proteomic study including sample preparation, data acquisition, and analysis, and edited the draft manuscript. JM assisted with the experimental design, evaluated the experimental results, edited the manuscript, and acquired funding. All authors contributed to the article and approved the submitted version.

## Conflict of Interest

The authors declare that the research was conducted in the absence of any commercial or financial relationships that could be construed as a potential conflict of interest.

## Publisher’s Note

All claims expressed in this article are solely those of the authors and do not necessarily represent those of their affiliated organizations, or those of the publisher, the editors and the reviewers. Any product that may be evaluated in this article, or claim that may be made by its manufacturer, is not guaranteed or endorsed by the publisher.

## Footnotes

1
www.ncbi.nlm.nih.gov

2
https://omics.pnl.gov/software/infernordn
