# Supplementary material for: Probing Light-Dependent Regulation of the Calvin Cycle Using a Multi-Omics Approach
Source: Front Plant Sci. 2021 Oct 4;12:733122. doi: 10.3389/fpls.2021.733122 (PMC8521058; doi:10.3389/fpls.2021.733122)
Supplement: Supplementary file 2 [file Data_Sheet_2.docx]

**Supplemental Figures**


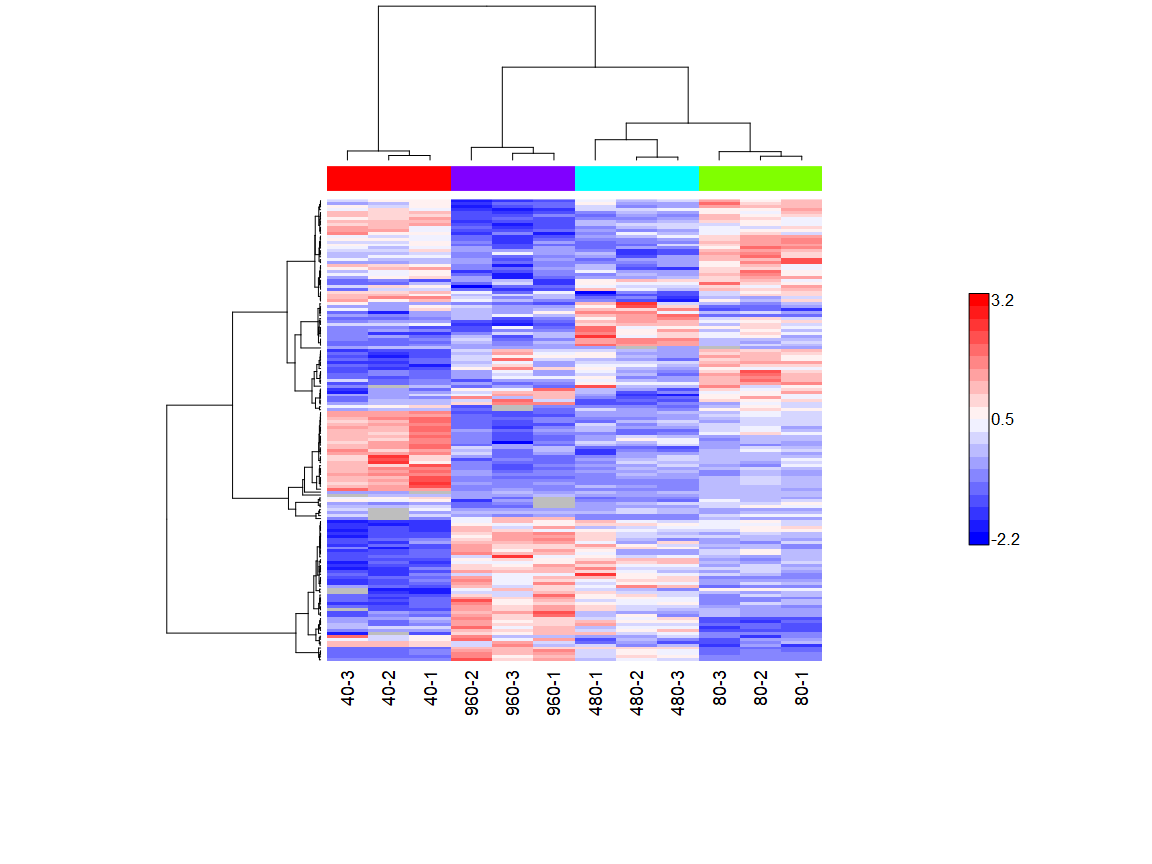


**Figure S1** Clustered proteomic heat map using Ward’s aggregation method and Pearson distance metric for filtered proteomic results (p < 0.05 and at least 2 data points per light level)


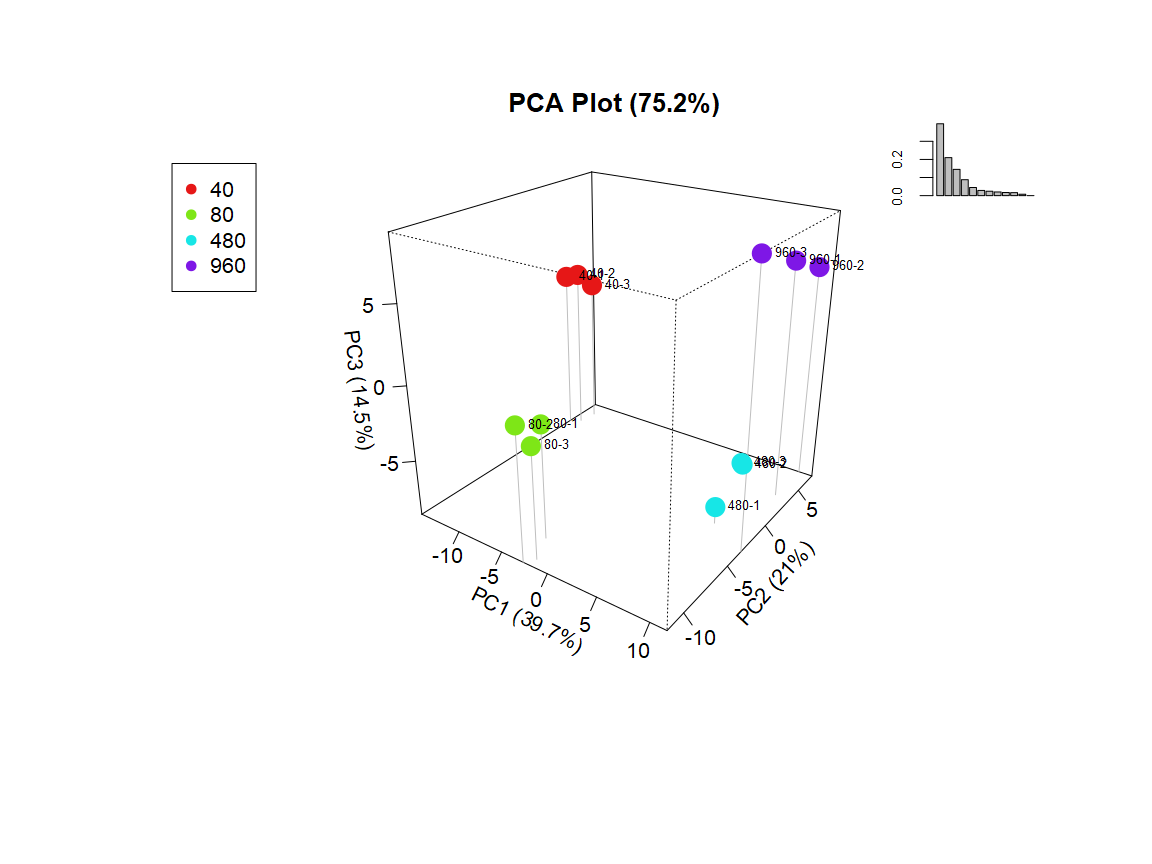


**Figure S2** Three-dimensional principal component analysis of proteomic data, demonstrating clustering of proteomic replicates


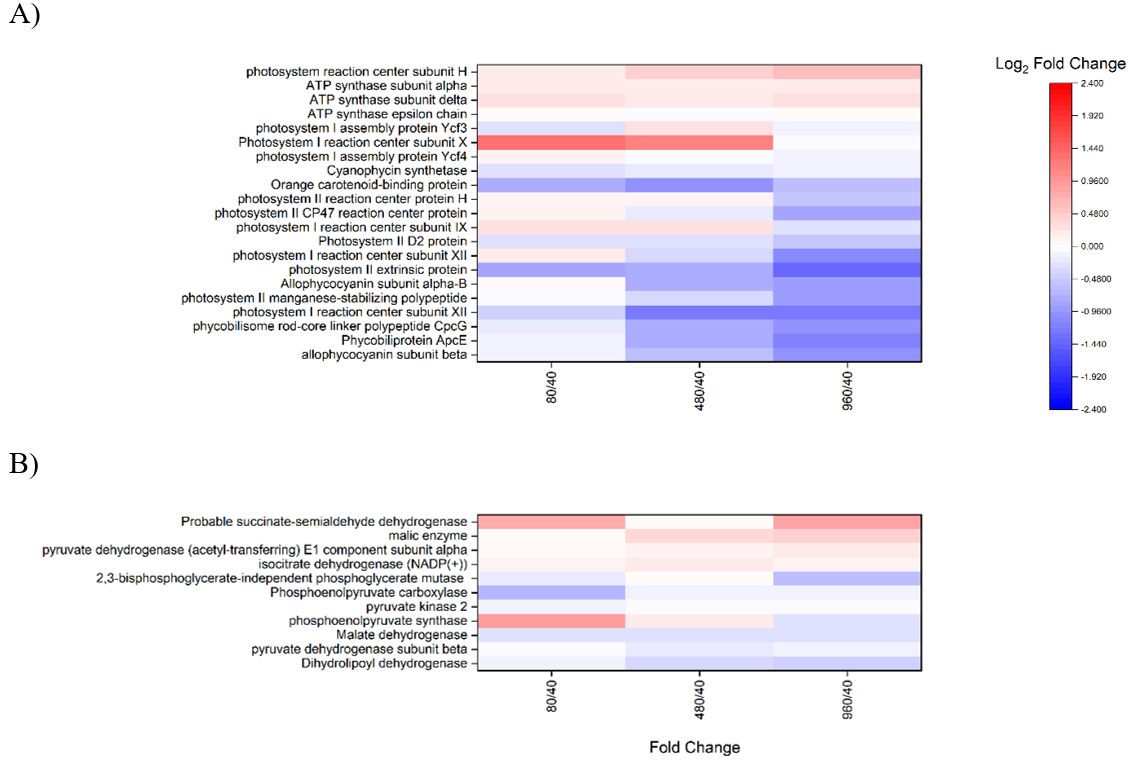


**Figure S3** Average proteomic fold-change results relative to the 40 µmol m^-2^ s^-1^ condition for selected a) photosystems, pigments and ATP synthase subunits, and b) TCA cycle related enzymes, n = 3 for each light condition


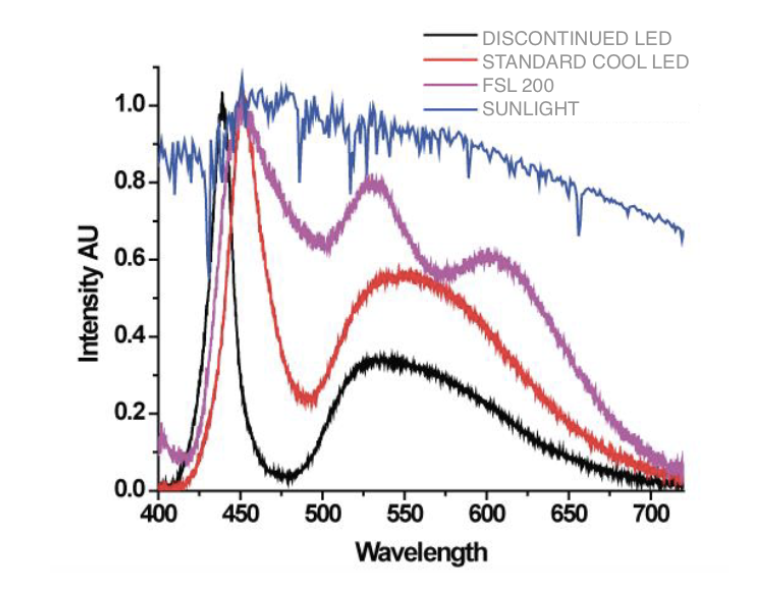


**Figure S4** Manufacturer-provided (PBR101, Phenometrics, MI) light spectra for the currently used cool LED (red), in comparison with sunlight (blue), older LED models (black), and full spectrum light LED (magenta)
